# Risk of chronic kidney disease in newly diagnosed SLE with preserved renal function: a national study

**DOI:** 10.1093/rheumatology/keag307

**Published:** 2026-06-16

**Authors:** Iftach Sagy, Itamar Ben Shitrit, Mahmoud Abu-Shakra, Lior Zeller, Amir Bieber, Keren Cohen-Hagai, Shaye Kivity, Oshrat E Tayer-Shifman

**Affiliations:** Lupus and Vasculitis Service, Department of Medicine, Cambridge University Hospitals NHS Foundation Trust, Cambridge, UK; Rheumatic Disease Unit, Soroka University Medical Center, Beer Sheva, Israel; School of Health Sciences, Ben Gurion University of the Negev, Beer Sheva, Israel; Clinical Research Center, Soroka University Medical Center, Beer Sheva, Israel; School of Health Sciences, Ben Gurion University of the Negev, Beer Sheva, Israel; Clinical Research Center, Soroka University Medical Center, Beer Sheva, Israel; Rheumatic Disease Unit, Soroka University Medical Center, Beer Sheva, Israel; School of Health Sciences, Ben Gurion University of the Negev, Beer Sheva, Israel; Rheumatic Disease Unit, Soroka University Medical Center, Beer Sheva, Israel; School of Health Sciences, Ben Gurion University of the Negev, Beer Sheva, Israel; Rappaport Faculty of Medicine, Technion Israel Institute of Technology, Haifa, Israel; Rheumatic Diseases Unit, Emek Medical Center, Afula, Israel; Department of Nephrology and Hypertension, Meir Medical Center, Kfar Saba, Israel; Gray Faculty of Medical & Health Sciences, Tel Aviv University, Tel Aviv, Israel; Gray Faculty of Medical & Health Sciences, Tel Aviv University, Tel Aviv, Israel; Rheumatology Unit, Meir Medical Center, Kfar Saba, Israel; Gray Faculty of Medical & Health Sciences, Tel Aviv University, Tel Aviv, Israel; Rheumatology Unit, Meir Medical Center, Kfar Saba, Israel

**Keywords:** SLE, chronic kidney disease, kidney outcome, LN

## Abstract

**Objectives:**

SLE is strongly associated with LN, a major cause of chronic kidney disease (CKD). However, long-term renal and cardiovascular outcomes in patients with SLE who have preserved kidney function and do not develop LN remain poorly characterized. We sought to investigate the development of CKD among newly diagnosed patients with SLE who had preserved kidney function and no evidence of concomitant LN at baseline, compared with matched control individuals.

**Methods:**

A national database study of newly diagnosed patients with SLE between 2015 and 2023. Patients with estimated glomerular filtration rate (eGFR) > 60 ml/min/1.73 m^2^ at diagnosis were matched to non-SLE controls by age, sex and ethnicity. Patients with LN were excluded. The primary outcome was incident CKD, defined as eGFR ≤ 60 ml/min/1.73 m^2^ after diagnosis. Secondary outcomes included end-stage kidney disease (ESKD), major adverse cardiovascular events (MACE) and all-cause mortality.

**Results:**

We identified 1145 patients with SLE and 91 681 matched controls, with a median follow-up of 5.77 years and similar baseline eGFR (103 vs 104 ml/min/1.73 m^2^). SLE was associated with a higher risk of CKD (5.2% vs 2.7%; HR 1.96, 95% CI 1.50–2.54), ESKD (HR 3.13, 95% CI 1.38–7.08), MACE (HR 1.63, 95% CI 1.31–2.04) and all-cause mortality (HR 4.52, 95% CI 3.71–5.50). Mean eGFR trajectories were similar between the groups. Diabetes (HR 1.51, 95% CI 1.39–1.64) and hypertension (HR 2.72, 95% CI 2.42–3.07) were the strongest risk factors for CKD and ESKD.

**Conclusion:**

Patients with SLE and preserved kidney function at diagnosis, without LN, are at increased risk of adverse renal outcomes, cardiovascular events and mortality, highlighting the importance of long-term monitoring and optimization of modifiable CKD risk factors.

Rheumatology key messages

- Patients with SLE without LN remain at increased risk of CKD and mortality.
- Traditional cardiovascular and metabolic risk factors substantially contribute to kidney disease progression in SLE.
- Long-term renal monitoring is important even in SLE patients with preserved kidney function at diagnosis.

## Introduction

SLE is a systemic autoimmune disease in which approximately half of patients develop kidney involvement, often manifested as LN [1]. According to the 2024 Kidney Disease: Improving Global Outcomes (KDIGO) guidelines of chronic kidney disease (CKD), LN is likely to result in CKD due to histologically detectable abnormalities that persist for >3 months [2]. CKD may manifest as either a sustained reduction in estimated glomerular filtration rate (eGFR) below 60 ml/min/1.73 m^2^ or as persistent albuminuria, reflecting residual kidney damage. By definition, either abnormality must be confirmed by at least two consecutive measurements separated by >3 months.

The risk of reduced kidney function, as reflected by a decline in eGFR, is observed in 30–50% of patients with LN over time, compared with 10–15% of patients with SLE without LN in long-term follow-up cohorts [3, 4]. End-stage kidney disease (ESKD) develops in ∼10% of patients with LN, and the presence of LN is an important risk factor for progression to both CKD and its associated adverse events, including accelerated atherosclerosis and cardiovascular events, ESKD and mortality over time [5].

Response to treatment initiation in LN is assessed by changes in proteinuria and serum creatinine over 6–12 months, according to the American College of Rheumatology and the European League Against Rheumatism (EULAR) guidelines [6, 7]. However, a recent study that followed 260 patients with LN for up to 35 years showed that eGFR at the end of the first year after LN diagnosis was more strongly associated with the long-term risk of CKD during subsequent follow-up than proteinuria [8].

Because LN is a major risk factor for CKD, most studies have not examined CKD trajectories in patients with SLE compared with non-SLE controls. In addition, CKD may arise from causes not directly related to SLE itself, such as hypertension or diabetes, which often result from SLE treatments such as calcineurin inhibitors and steroids. Therefore, we aim to evaluate the risk of CKD in a subgroup of newly diagnosed SLE patients with preserved eGFR and without concurrent LN, compared with matched controls.

## Methods

### Study population

Patients with SLE were identified from the Clalit Health Services (CHS) SLE registry. CHS is the largest health maintenance organization in Israel, covering >54% of the national population. It operates 14 hospitals and >1600 outpatient clinics and uses comprehensive, real-time electronic medical records that capture demographic data, diagnoses, hospitalizations, laboratory results and medication dispensing through CHS pharmacies. The study was approved by the CHS Central Ethics Committee (COM-17–0212), which granted a waiver of informed consent.

Patients with SLE and LN were identified from the CHS database using established case-identification methods applied to the CHS SLE registry. As the full methodology has been described previously [9], we included adults aged 18 years or older with an ICD-9 diagnosis code for SLE recorded by a rheumatologist, along with supporting evidence of disease, defined as either a documented ANA result or continuous HCQ treatment for at least 3 months.

Within the SLE cohort, LN was defined as documented kidney biopsy or exposure to LN-directed therapy for at least 3 months, in conjunction with evidence of renal involvement, including a recorded diagnosis of CKD or ESKD or proteinuria.

### Data extraction and study outcomes

We included patients from the CHS SLE registry who were diagnosed with SLE between 2015 and 2023 (Supplementary Fig. S1). The date of the first SLE diagnosis was defined as the index date. Patients with SLE were then matched to non-SLE controls, as described below. Inclusion required preserved kidney function at baseline, defined as an estimated eGFR ≥ 60 ml/min/1.73 m^2^, calculated using the CKD-EPI formula [10]. Patients were required to have at least one eGFR measurement in the year preceding the index date and at least one eGFR measurement in each year thereafter. When multiple values were available within a given year, the mean annual eGFR was calculated. We excluded patients with a concurrent diagnosis of LN from the index date through the end of follow-up.

The primary outcome was incident CKD, defined as at least two eGFR measurements < 60 ml/min/1.73 m^2^, separated by >90 days, during follow-up. When multiple measurements were available within a given year, an annual mean eGFR value < 60 ml/min/1.73 m^2^ was used. Secondary outcomes included incident major adverse cardiovascular events (MACE) and ESKD, as well as all-cause mortality. MACE was identified using ICD-9 codes for myocardial infarction, coronary revascularization and stroke. ESKD was identified using ICD-9 codes for renal replacement therapy, including kidney transplantation.

### Statistical analysis

Baseline characteristics were summarized using means (S.D.) for continuous variables and frequencies (percentages) for categorical variables. Group comparisons used Student’s *t*-tests for continuous variables and *χ*^2^ tests for categorical variables. Standardized mean differences (SMDs) were used to assess balance between groups; SMD < 0.10 indicated adequate balance.

Each SLE patient was matched 1:100 to controls without SLE on birth year, sex and ethnicity. Controls were assigned the same index date as their matched SLE patient (the SLE diagnosis date). After matching, exclusions were applied only based on measurements relative to the index date: (1) no eGFR measurement within ±365 days of the index, (2) eGFR < 60 ml/min/1.73 m^2^ before the index date or (3) urine protein creatinine ratio (UPCR) >500 mg/g or urine albumin creatinine ratio (ACR) >300 mg/g before the index.

The Cox proportional hazards models estimated hazard ratios (HRs) and 95% CIs for the association between SLE and CKD. Crude models were constructed. Adjusted models controlled for age at index, sex, baseline eGFR, BMI (<30 vs ≥30 kg/m^2^), smoking status, diabetes and hypertension. Kaplan–Meier curves with log-rank tests were used to visualize event-free survival. The same approach was used for secondary outcomes (ESKD, MACE, all-cause mortality). To account for death as a competing event for non-fatal outcomes (CKD, ESKD, MACE), a competing risks analysis was conducted. Cause-specific Cox models treated death as censoring, while Fine-Gray subdistribution hazard models accounted for death as a competing risk. Both crude and adjusted models were fitted, with adjustment for the same covariates as the primary analysis.

### Subgroup and sensitivity analysis

Subgroup analyses were conducted separately within the lupus and control cohorts to assess risk factor effects on CKD development, including age (<60 vs ≥60 years), sex, socioeconomic status (high/medium vs low), diabetes, hypertension and BMI (<30 vs ≥30 kg/m^2^). Time-varying Cox models with clustered standard errors were used to evaluate medication effects (RAAS inhibitors, prednisone and HCQ) on CKD risk among patients receiving treatment before CKD diagnosis, with binary exposure (any vs none) for RAAS inhibitors and cumulative dose for prednisone and HCQ. All models were adjusted for age, sex, baseline eGFR, diabetes and hypertension.

A sensitivity analysis restricted to patients with ≥1 urine protein measurement after the index date evaluated the combined outcome of CKD, defined as both eGFR < 60 ml/min/1.73 m^2^ (confirmed) and UPCR > 500 mg/g or ACR >  300 mg/g. Cox models (crude and adjusted) were estimated to assess associations between lupus and all study outcomes (CKD, ESKD, MACE, mortality) in this subpopulation. All statistical analyses were performed using R version 4.3.2 (R Foundation for Statistical Computing, Vienna, Austria).

## Results

We matched 91 681 controls to a cohort of 1145 patients with SLE. The median follow-up was 5.77 years (3.56–8.32) for the entire cohort (Supplementary Fig. S1). Baseline characteristics, including sex, socioeconomic status, smoking status and multiple comorbidities, were well balanced between the groups (Table 1). At the index date, baseline eGFR was similar between the groups (103 vs 104 ml/min/1.73 m^2^; SMD = −0.04). At baseline, 79% of patients with SLE had received HCQ, and 19% had received biologics.

**Table 1:** Baseline characteristics.

Characteristic	*N*	**Control, *N* = 91 681** ^a^	**Lupus, *N* = 1145** ^a^	**Difference** ^b^	***P*-value** ^c^
Age at index (years)	92 826	47 (16)	45 (16)	0.14	<0.001
Sex	92 826			0.03	0.3
Female		79 620 (87%)	982 (86%)		
Male		12 061 (13%)	163 (14%)		
Socioeconomic status	86 162			0.08	0.040
Low		22 522 (26%)	294 (28%)		
Medium		47 445 (56%)	610 (57%)		
High		15 134 (18%)	157 (15%)		
Jewish ethnicity	60 977	39 422 (65%)	432 (61%)	0.10	0.006
BMI (kg/m²)	90 767	26.9 (6.1)	26.1 (5.9)	0.13	<0.001
Ever smoker	92 826	15 155 (17%)	218 (19%)	−0.07	0.023
Diabetes mellitus	92 826	17 940 (20%)	176 (15%)	0.11	<0.001
Congestive heart failure	92 826	2995 (3.3%)	77 (6.7%)	−0.16	<0.001
Antiphospholipid syndrome	92 826	280 (0.3%)	158 (14%)	0.55	<0.001
Dyslipidaemia	92 826	45 943 (50%)	470 (41%)	0.18	<0.001
Cerebrovascular accident	92 826	5055 (5.5%)	102 (8.9%)	−0.13	<0.001
Ischemic heart disease	92 826	10 977 (12%)	219 (19%)	−0.20	<0.001
Hypertension	92 826	27 738 (30%)	367 (32%)	−0.04	0.2
Baseline eGFR (ml/min/1.73 m^2^)	92 826	103 (18)	104 (21)	−0.04	0.2
HCQ	N/A	N/A	905 (79%)	N/A	N/A
AZA	N/A	N/A	259 (23%)	N/A	N/A
MMF	N/A	N/A	63 (5.5%)	N/A	N/A
MTX	N/A	N/A	291 (25%)	N/A	N/A
Prednisone	N/A	N/A	713 (62%)	N/A	N/A
Biologics (rituximab or belimumab)	N/A	N/A	212 (19%)	N/A	N/A
Calcineurin inhibitors	N/A	N/A	45 (3.9%)	N/A	N/A
RAAS inhibitors	92 826	24 315 (27%)	298 (26%)	0.01	0.7
Statins	92 826	35 440 (39%)	365 (32%)	0.14	<0.001
Aspirin	92 826	472 (0.5%)	7 (0.6%)	−0.01	0.7
Antihypertensives (BB/CCB/diuretics)	92 826	34 864 (38%)	529 (46%)	−0.17	<0.001
Diabetes medications	92 826	20 775 (23%)	219 (19%)	0.09	0.005

a *n* (%); mean (S.D.).

b Standardized mean difference.

c Pearson’s *χ*^2^*d* test; Welch two sample *t*-test; Fisher’s exact test.

eGFR, estimated glomerular filtration rate; RAAS, renin–angiotensin–aldosterone system; BB, beta blockers; CCB, calcium channel blockers.

**Figure 1:**
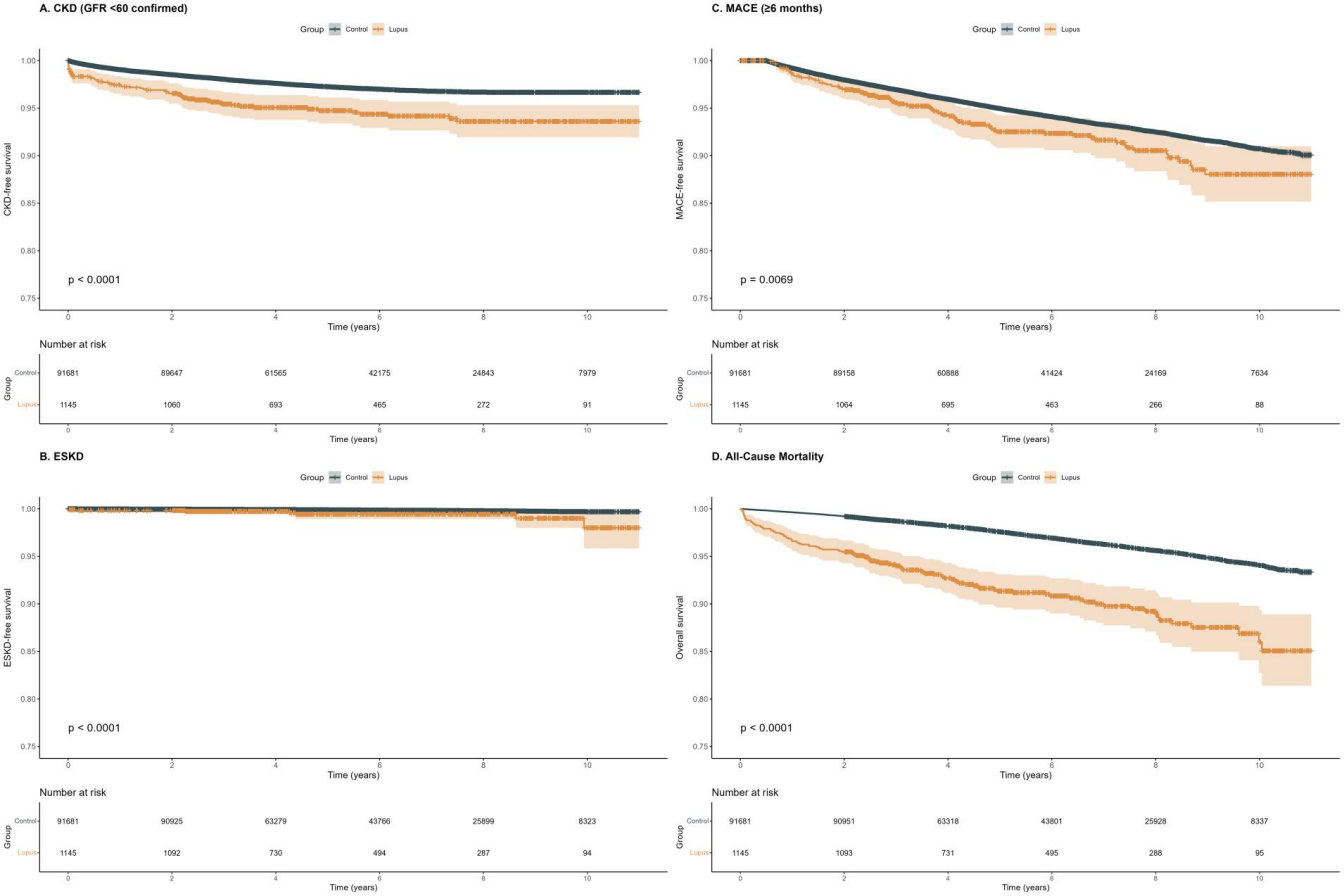
Risk of adverse outcomes in patients with SLE and preserved kidney function. Kaplan–Meier curves comparing patients with SLE and matched controls for (**A**) incident chronic kidney disease (CKD; confirmed estimated glomerular filtration rate [eGFR] < 60 mL/min/1.73 m^2^), (**B**) end-stage kidney disease (ESKD), (**C**) major adverse cardiovascular events (MACE) and (**D**) all-cause mortality. Log-rank *P* values are shown. Numbers at risk are presented below each panel

Study outcomes are presented in Table 2 and Fig. 1. During follow-up, patients with SLE had a higher risk of developing CKD, with 60 patients (5.2%) developing CKD compared with 2507 controls (2.7%) (HR 1.96, 95% CI 1.50–2.54). Seven patients with SLE (0.6%) developed ESKD compared with 137 controls (0.1%) (HR 3.13, 95% CI 1.38–7.08). The SLE group also had a higher risk of MACE (HR 1.63, 95% CI 1.31–2.04) and all-cause mortality (HR 4.52, 95% CI 3.71–5.50). Having a medical background of smoking (HR 1.16, 95% CI 1.06–1.28), diabetes (HR 1.51, 95% CI 1.39–1.64) and hypertension (HR 2.72, 95% CI 2.42–3.07) were the strongest factors associated with CKD and ESKD (Supplementary materials).

**Table 2:** Study outcomes.

	Frequencies			Crude analysis			Adjusted		
Outcome	**Control, *N* = 91** **681**^a^	**Lupus, *N* = 1145** ^a^	***P*-value** ^a^	**HR (95% CI)** ^b^	**95% CI** ^b^	*P*-value	**HR (95% CI)** ^b^	**95% CI** ^b^	*P*-value
CKD (GFR <60)	2507 (2.7%)	60 (5.2%)	<0.001	2.05	1.58, 2.64	<0.001	1.96	1.50, 2.54	<0.001
ESKD	137 (0.1%)	7 (0.6%)	0.002	4.41	2.06, 9.42	<0.001	3.13	1.38, 7.08	0.006
MACE	5308 (5.8%)	83 (7.2%)	0.036	1.35	1.08, 1.67	0.007	1.63	1.31, 2.04	<0.001
Mortality	2998 (3.3%)	106 (9.3%)	<0.001	3.04	2.51, 3.69	<0.001	4.52	3.71, 5.50	<0.001

a Pearson’s *χ*^2^*d* test; Fisher’s exact test, *n* (%).

b HR, hazard ratio.

**Figure 2:**
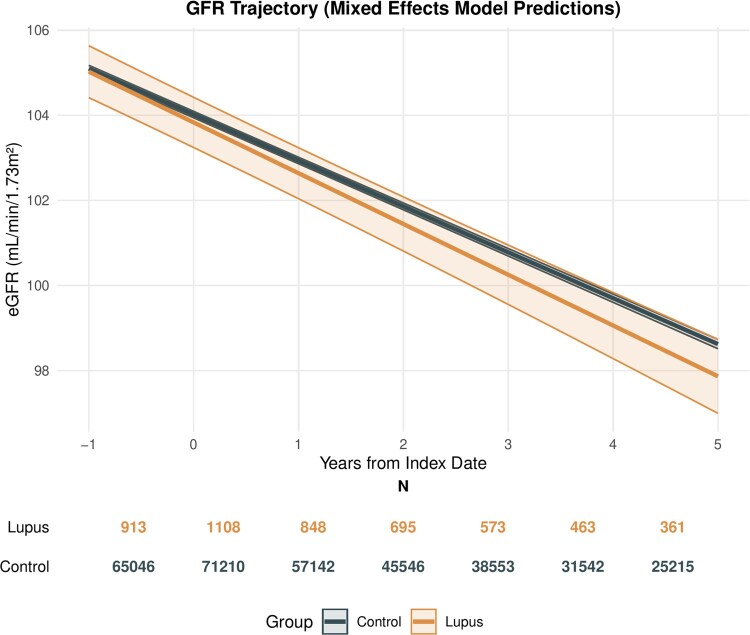
Trajectory of kidney function over time. Mean predicted eGFR trajectories over time in patients with SLE and matched controls, estimated using mixed-effects models. Both the groups had similar baseline eGFR and comparable rates of change during follow-up. eGFR, estimated glomerular filtration rate

We conducted a sensitivity analysis restricted to patients with available urine protein measurements after the index diagnosis, available for 801 (69.9%) patients with SLE and 31 971 (34.9%) controls, using a combined renal outcome defined as eGFR < 60 ml/min/1.73 m^2^ and UPCR > 500 mg/g (Supplementary materials). In this analysis, SLE remained associated with a higher risk of CKD (HR 1.94, 95% CI 1.06–3.53), ESKD (HR 2.55, 95% CI 1.03–6.34) and all-cause mortality (HR 3.74, 95% CI 2.81–4.98). Changes in the mean eGFR over time are shown in Fig. 2. Both the groups had similar mean eGFR at the index date and at the last follow-up, with comparable mean changes over time (Supplementary materials).

**Figure 3:**
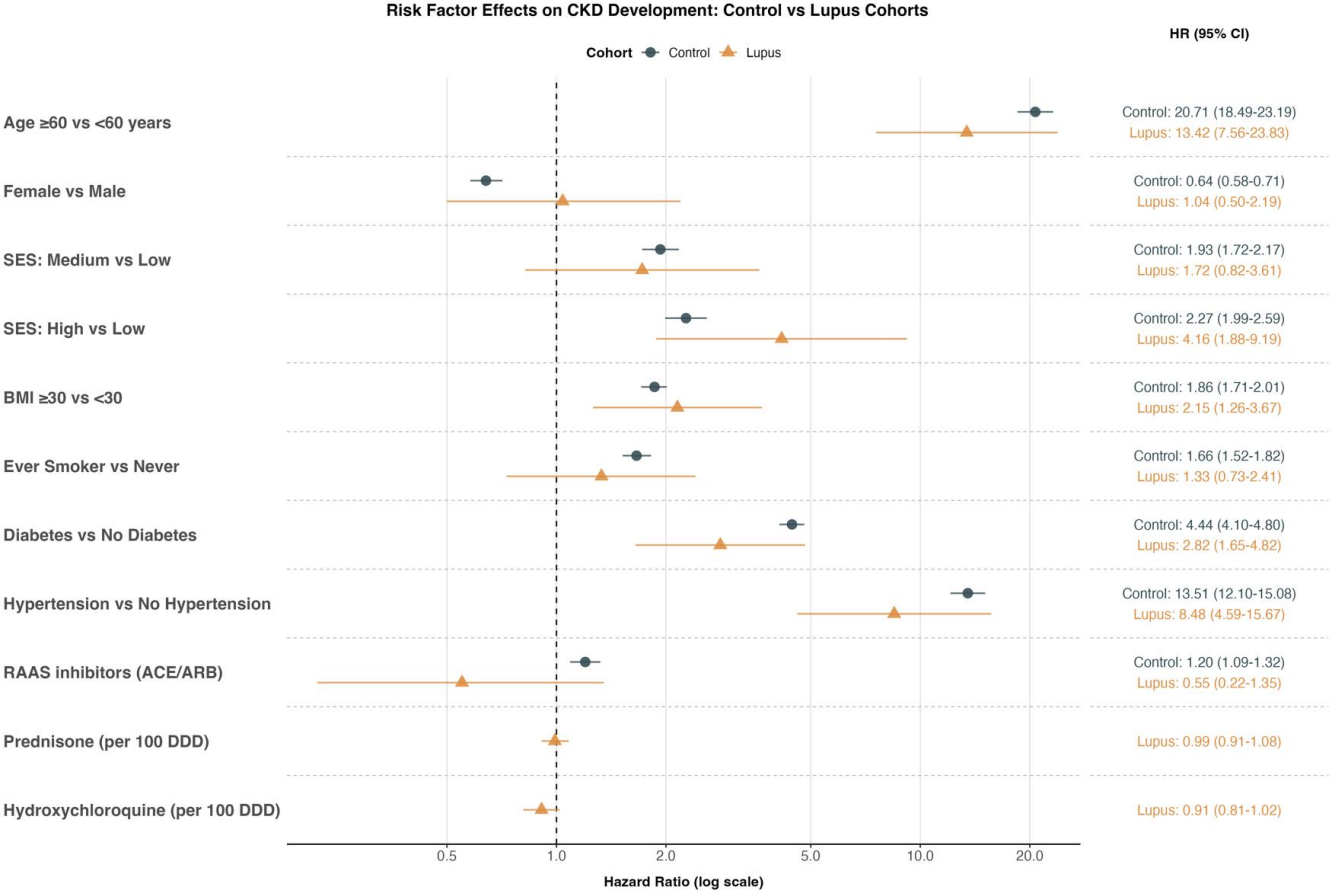
Risk factors for chronic kidney disease in lupus and control cohorts. Forest plot showing hazard ratios (HRs) and 95% CIs for risk factors associated with incident chronic kidney disease (CKD) in patients with SLE and matched controls. Models were adjusted for age, sex, baseline estimated glomerular filtration rate (eGFR), diabetes and hypertension. SES, socioeconomic status; RAAS, renin–angiotensin–aldosterone system; ACE, angiotensin-converting enzyme; ARB, angiotensin receptor blocker; DDD, defined daily dose

Subgroup analyses are shown in Fig. 3. Age older than 60 years, higher socioeconomic status, BMI > 30, diabetes and hypertension were associated with increased risk of CKD among both SLE patients and controls. Among patients with SLE, use of prednisone and HCQ was not associated with an increased risk of CKD; HCQ showed a marginal, non-statistically significant association with a lower risk of CKD (HR 0.91, 95% CI 0.81–1.02).

## Discussion

Our main findings are that patients with SLE without a diagnosis of LN who have preserved renal function at diagnosis are at increased risk of subsequent decline in renal function (eGFR < 60 ml/min/1.73 m^2^), ESKD, MACE and all-cause mortality compared with matched controls. These findings remained consistent across sensitivity analyses using CKD definitions based on both eGFR < 60 ml/min/1.73 m^2^ and proteinuria.

Historically, kidney disease was a leading cause of mortality among patients with SLE, and despite advanced therapeutic approaches, developing CKD is still a major concern in patients with SLE and an important contributor to both morbidity and mortality. A recent study reported that CKD-attributed mortality among patients with SLE increased in the USA between 1999 and 2020 [11]. Moreover, SLE patients with eGFR < 60 ml/min/1.73 m^2^ had a higher risk of cardiovascular disease, ESKD, and septic shock in a French national study [12]. In these studies, among many others, LN was identified as an important risk factor for adverse outcomes, including significant morbidity and mortality, based on comparisons between patients with SLE and LN and those with SLE without LN. For instance, a diagnosis of LN class IV was associated with an increased long-term risk of developing CKD stages 3–5 in an inception cohort followed for >16 years [13]. Similarly, an inception cohort of 175 patients with SLE, followed for 18.3 years, found that a diagnosis of LN was the strongest risk factor for the long-term development of CKD [14]. Among the proposed reasons for LN’s impact on CKD risk are lower baseline eGFR and higher albuminuria, a higher prevalence of background comorbidities such as hypertension, lower complement levels at diagnosis and poorer treatment response rates [15, 16].

Intriguingly, there are very few studies comparing the risk of CKD in patients with SLE without LN with that of the general population, in contrast to the extensive literature comparing CKD risk between SLE patients with and without LN. The global age-standardized prevalence of CKD in adults was 14.2% in 2023, representing an increase of 3.5% since 1990, and CKD was the ninth leading cause of death worldwide [17]. Traditional CKD risk factors, including hypertension, obesity and diabetes, are more prevalent among patients with SLE, in some studies occurring at rates two to three times higher than in non-SLE comparators [18]. This suggests that LN may obscure the contribution of other factors to the overall increased risk of CKD among patients with SLE. Only a single national study from Korea by Choi *et al.* compared patients with SLE, regardless of LN status, with the general population to evaluate the risk of ESKD [19]. The main finding was that SLE was associated with a higher risk of ESKD compared with non-SLE controls across all subgroups. Although this was the first large-scale study to compare renal outcomes between patients with SLE and the general population, it had several important limitations. SLE cases were identified solely using ICD-10 codes without additional validation to ensure diagnostic accuracy. The primary outcome was ESKD, which is far less common than CKD. The analysis excluded patients with newly diagnosed SLE, and the cohort was assembled between 2008 and 2013, a period when biologic therapies were rarely used in routine SLE management.

The findings that patients with SLE without overt LN at diagnosis remain at increased risk of developing CKD, cardiovascular events and increased mortality rates, despite treatment with conventional immunosuppressive therapies and biologic agents, suggest that mechanisms beyond immune-mediated renal injury contribute to these adverse outcomes. These mechanisms appear to share a common pathophysiology involving atherosclerosis and cardiometabolic disturbances. Accumulating evidence highlights accelerated atherosclerosis as a central underlying process linking renal, cardiovascular and survival risks in this population, emphasizing the pivotal role of vascular disease in the morbidity and mortality of SLE, even in the absence of clinically apparent LN [20, 21].

Identifying risk factors for long-term CKD in patients with SLE is particularly important around the time of initial diagnosis. Two recently published studies reported that even a mild reduction in eGFR (<80 ml/min/1.73 m^2^) during the first year after SLE diagnosis was associated with an increased risk of developing CKD later in the disease course. The subsequent development of CKD is a significant risk factor for later adverse outcomes, including not only ESKD but also a higher likelihood of cardiovascular disease, hospitalizations due to SLE flares, severe infections and overall mortality [14, 22]. Moreover, this risk remains higher than that for other causes of immune-mediated glomerulonephritis, such as anti-neutrophil cytoplasmic antibody-associated vasculitis [23].

We acknowledge several important limitations of our study. First, this was a retrospective analysis, and therefore, causality cannot be assumed. Second, as this national cohort represents the Israeli SLE population, differences in the prevalence of CKD risk factors and in access to specific therapies may limit the generalizability of our findings to other regions. Notably, the Israeli healthcare system provides universal medical coverage with broad access to biologic therapies and CVD medications, which may contribute to a relatively high life expectancy compared with other OECD countries. Third, because systematic urine protein measurements were lacking in a substantial proportion of patients, we focused primarily on an eGFR decline below 60 ml/min/1.73 m^2^ rather than applying the full CKD definition used in the literature, which also includes persistent proteinuria. However, given that our cohort consisted of patients with SLE without a history of LN and who did not develop LN during follow-up, this approach is clinically reasonable. Fourth, we included only patients with sufficient eGFR data in the year preceding SLE diagnosis and throughout follow-up, which may have introduced selection bias by preferentially including individuals with more complete longitudinal laboratory data. Finally, because of the nature and scale of this database analysis, we did not have access to standardized measures of SLE disease activity (such as SLEDAI-2K scores) or to detailed data on disease flares, and we were also unable to ascertain specific causes of CKD, such as those derived from kidney biopsy reports.

Our analysis has several strengths. First, our cohort was assembled from a national population of patients with validated SLE diagnoses, and, unlike most previous studies, we compared patients with SLE, regardless of LN status, with matched controls. This approach better reflects the real-world magnitude of CKD risk among patients with SLE. Second, our analysis included only newly diagnosed patients with SLE and a relatively long follow-up period, thereby minimizing the influence of later-developing CKD risk factors, such as diabetes and hypertension, as well as the cumulative impact of LN-related kidney damage. Finally, we restricted the analysis to patients with preserved baseline eGFR, further emphasizing that the risk of CKD is not confined to patients with LN.

## Conclusion

Our findings suggest that, in a large national cohort of patients with SLE and preserved renal function who did not have LN, there was an increased risk of adverse kidney outcomes, cardiovascular morbidity and mortality. Patients with SLE had a higher risk of developing CKD and ESKD, as well as an increased risk of MACE and all-cause mortality, compared with matched controls. These findings may indicate underdiagnosis of LN and highlight the importance of addressing traditional CKD risk factors when treating patients with SLE. Thus, the clinical implications of this analysis for both rheumatologists and general practitioners include the need for early recognition and timely referral of patients with SLE, particularly in the era of emerging CKD treatment options.

## Supplementary Material

keag307_Supplementary_Data

## Data Availability

The data supporting this study were obtained from Clalit Health Services (CHS) and are not publicly available due to patient privacy and institutional data protection restrictions. Access to de-identified data may be considered by the corresponding author upon reasonable request and subject to approval by Clalit Health Services and the relevant ethics committees.
