# Supplementary material for: Risk of chronic kidney disease in newly diagnosed SLE with preserved renal function: a national study
Source: Rheumatology (Oxford). 2026 Jun 16;65(7):keag307. doi: 10.1093/rheumatology/keag307 (PMC13318493; doi:10.1093/rheumatology/keag307)

Contents

[1. Primary analysis 2](#_Toc220580232)

[1.1. Primary analysis - multivariable regression models 2](#_Toc220580233)

[1.1.1. Table SXA: Multivariable Cox Regression - CKD 2](#_Toc220580234)

[1.1.2. Table SXB: Multivariable Cox Regression - ESKD 2](#_Toc220580235)

[1.1.3. Table SXC: Multivariable Cox Regression - MACE 3](#_Toc220580236)

[1.1.4. Table SXD: Multivariable Cox Regression - All-Cause Mortality 3](#_Toc220580237)

[1.2. Table SX Mixed Effects Model Results for Kidney Function Trajectories 4](#_Toc220580238)

[1.3. Table SX: Medication Effects on CKD Within Each Cohort 5](#_Toc220580239)

[1.4. Table Sx: Risk Factor Effects on CKD Within Each Cohort 5](#_Toc220580240)

[2. Sensitivity analysis 5](#_Toc220580241)

[2.1. Competing Risks Analysis – Full Cohort 5](#_Toc220580242)

[2.1. Table SX Baseline Characteristics analysis – Subpopulation with at least one protein tests after index 7](#_Toc220580243)

[2.2. Outcome analysis – Subpopulation with at least one protein tests after index 8](#_Toc220580244)

[2.2.1. Table SX: Cox Regression 8](#_Toc220580245)

[2.3. Multivariable models - Subpopulation 8](#_Toc220580246)

[2.3.1. *Table SX*A: Multivariable Cox Regression - CKD (per GFR<60 AND Protein>500) - Subpopulation 8](#_Toc220580247)

[2.3.2. Table S*X*B: Multivariable Cox Regression - ESKD - Subpopulation 9](#_Toc220580248)

[2.3.3. Table S*X*C: Multivariable Cox Regression - MACE - Subpopulation 10](#_Toc220580249)

[2.3.4. Table S*X*D: Multivariable Cox Regression - All-Cause Mortality - Subpopulation 10](#_Toc220580250)

3. Study flow chart (Supplementary Figure S1)………………………………………………….…11

4. Proteinuria trajectory graph (Supplementary Figure S2)…………………………………..12

5. Interaction between SLE and traditional risk factors for CKD risk (supplementary Figure S3)………………………………………………………………………………………………………….13

# Primary analysis

## Primary analysis - multivariable regression models

### Table SXA: Multivariable Cox Regression - CKD

| **Variable** | **N** | **Event N** | **HR^1^** | **95% CI^1^** | **P-value** |
| --- | --- | --- | --- | --- | --- |
| **Lupus (vs Control)** | 90,767 | 2,503 | 1.96 | 1.50, 2.54 | **<0.001** |
| **Female sex** |  |  |  |  |  |
| Female | 78,844 | 2,025 | — | — |  |
| Male | 11,923 | 478 | 0.99 | 0.90, 1.10 | >0.9 |
| **Age at index (years)** | 90,767 | 2,503 | 1.05 | 1.04, 1.05 | **<0.001** |
| **Baseline eGFR (mL/min/1.73m²)** | 90,767 | 2,503 | 0.93 | 0.93, 0.93 | **<0.001** |
| **Body mass index (kg/m²)** | 90,767 | 2,503 | 1.01 | 1.00, 1.01 | **0.020** |
| **Ever smoker** | 90,767 | 2,503 | 1.16 | 1.06, 1.28 | **0.002** |
| **Diabetes mellitus** | 90,767 | 2,503 | 1.51 | 1.39, 1.64 | **<0.001** |
| **Hypertension** | 90,767 | 2,503 | 2.72 | 2.42, 3.07 | **<0.001** |
| ^1^HR = Hazard Ratio, CI = Confidence Interval | | | | | |

### Table SXB: Multivariable Cox Regression - ESKD

| **Variable** | **N** | **Event N** | **HR^1^** | **95% CI^1^** | **P-value** |
| --- | --- | --- | --- | --- | --- |
| **Lupus (vs Control)** | 90,767 | 135 | 3.13 | 1.38, 7.08 | **0.006** |
| **Female sex** |  |  |  |  |  |
| Female | 78,844 | 99 | — | — |  |
| Male | 11,923 | 36 | 1.27 | 0.85, 1.88 | 0.2 |
| **Age at index (years)** | 90,767 | 135 | 0.97 | 0.95, 0.98 | **<0.001** |
| **Baseline eGFR (mL/min/1.73m²)** | 90,767 | 135 | 0.95 | 0.94, 0.96 | **<0.001** |
| **Body mass index (kg/m²)** | 90,767 | 135 | 0.99 | 0.96, 1.02 | 0.4 |
| **Ever smoker** | 90,767 | 135 | 1.39 | 0.95, 2.04 | 0.091 |
| **Diabetes mellitus** | 90,767 | 135 | 3.24 | 2.21, 4.75 | **<0.001** |
| **Hypertension** | 90,767 | 135 | 8.93 | 4.88, 16.3 | **<0.001** |
| ^1^HR = Hazard Ratio, CI = Confidence Interval | | | | | |

### Table SXC: Multivariable Cox Regression - MACE

| **Variable** | **N** | **Event N** | **HR^1^** | **95% CI^1^** | **P-value** |
| --- | --- | --- | --- | --- | --- |
| **Lupus (vs Control)** | 90,767 | 5,319 | 1.63 | 1.31, 2.04 | **<0.001** |
| **Female sex** |  |  |  |  |  |
| Female | 78,844 | 3,777 | — | — |  |
| Male | 11,923 | 1,542 | 2.11 | 1.98, 2.24 | **<0.001** |
| **Age at index (years)** | 90,767 | 5,319 | 1.05 | 1.04, 1.05 | **<0.001** |
| **Baseline eGFR (mL/min/1.73m²)** | 90,767 | 5,319 | 1.00 | 1.00, 1.00 | 0.3 |
| **Body mass index (kg/m²)** | 90,767 | 5,319 | 1.01 | 1.00, 1.01 | **<0.001** |
| **Ever smoker** | 90,767 | 5,319 | 1.44 | 1.35, 1.53 | **<0.001** |
| **Diabetes mellitus** | 90,767 | 5,319 | 1.68 | 1.59, 1.78 | **<0.001** |
| **Hypertension** | 90,767 | 5,319 | 2.52 | 2.35, 2.71 | **<0.001** |
| ^1^HR = Hazard Ratio, CI = Confidence Interval | | | | | |

### Table SXD: Multivariable Cox Regression - All-Cause Mortality

| **Variable** | **N** | **Event N** | **HR^1^** | **95% CI^1^** | **P-value** |
| --- | --- | --- | --- | --- | --- |
| **Lupus (vs Control)** | 90,767 | 3,006 | 4.52 | 3.71, 5.50 | **<0.001** |
| **Female sex** |  |  |  |  |  |
| Female | 78,844 | 2,328 | — | — |  |
| Male | 11,923 | 678 | 1.35 | 1.24, 1.47 | **<0.001** |
| **Age at index (years)** | 90,767 | 3,006 | 1.11 | 1.10, 1.11 | **<0.001** |
| **Baseline eGFR (mL/min/1.73m²)** | 90,767 | 3,006 | 1.00 | 1.00, 1.01 | **0.004** |
| **Body mass index (kg/m²)** | 90,767 | 3,006 | 1.00 | 0.99, 1.00 | 0.3 |
| **Ever smoker** | 90,767 | 3,006 | 1.41 | 1.30, 1.54 | **<0.001** |
| **Diabetes mellitus** | 90,767 | 3,006 | 1.38 | 1.28, 1.49 | **<0.001** |
| **Hypertension** | 90,767 | 3,006 | 1.25 | 1.14, 1.37 | **<0.001** |
| ^1^HR = Hazard Ratio, CI = Confidence Interval | | | | | |

## Table SX Mixed Effects Model Results for Kidney Function Trajectories

Results from linear mixed effects models examining longitudinal changes in eGFR and proteinuria. Both models include random intercepts and slopes (when convergent) to account for within-patient correlation. The Time × Lupus interaction term is the key parameter testing whether lupus patients have different rates of kidney function change compared to controls.

| **Outcome** | **Fixed Effect** | **Estimate** | **95% CI** | **P-value** |  |
| --- | --- | --- | --- | --- | --- |
| eGFR | Intercept | 100.61 | [100.41, 100.81] | <0.001 | * |
|  | Time (years) | -1.08 | [-1.10, -1.06] | <0.001 | * |
|  | Lupus (vs Control) | -0.18 | [-0.78, 0.41] | 0.542 |  |
|  | Age (centered) | -0.88 | [-0.89, -0.88] | <0.001 | * |
|  | Female (vs Male) | 3.41 | [3.19, 3.62] | <0.001 | * |
|  | Time × Lupus | -0.11 | [-0.25, 0.02] | 0.095 |  |
| log(Proteinuria) | Intercept | 4.898 | [4.771, 5.026] | <0.001 | * |
|  | Time (years) | 0.008 | [-0.012, 0.029] | 0.438 |  |
|  | Lupus (vs Control) | -0.148 | [-0.291, -0.004] | 0.044 | * |
|  | Age (centered) | -0.009 | [-0.011, -0.006] | <0.001 | * |
|  | Female (vs Male) | -0.218 | [-0.344, -0.093] | <0.001 | * |
|  | Time × Lupus | -0.017 | [-0.062, 0.028] | 0.459 |  |
| Mixed effects models with random intercepts and slopes (if convergent) | | | | | |
| Models adjusted for age (centered) and gender | | | | | |
| * P < 0.05 | | | | | |
| Time × Lupus interaction tests differential rate of change between groups | | | | | |
| eGFR random effects: SD(Intercept)=9.68, SD(Slope)=1.43, Residual=7.48 | | | | | |
| eGFR random effects: SD(Intercept)=0.04, SD(Slope)=1.43, Residual=7.48 | | | | | |
| Proteinuria random effects: SD(Intercept)=0.913, SD(Slope)=0.189, Residual=0.615 | | | | | |
| Proteinuria random effects: SD(Intercept)=-0.433, SD(Slope)=0.189, Residual=0.615 | | | | | |
| eGFR model fit: AIC=4551316.3, BIC=4551429.9, N(patients)=83288, N(observations)=628816 | | | | | |
| Proteinuria model fit: AIC=15965.3, BIC=16032.9, N(patients)=1830, N(observations)=6410 | | | | | |

## Table SX: Medication Effects on CKD Within Each Cohort

|  |  | **Control Cohort** | | **Lupus Cohort** | |
| --- | --- | --- | --- | --- | --- |
| **Medication** | **Exposure Type** | **HR (95% CI)** | **P-value** | **HR (95% CI)** | **P-value** |
| **RAAS (ACE/ARB)** | Binary | 1.20 (1.09-1.32) | <0.001 | 0.55 (0.22-1.35) | 0.191 |
| **Prednisone** | Per 100 DDD | -- | -- | 0.99 (0.91-1.08) | 0.865 |
| **Hydroxychloroquine** | Per 100 DDD | -- | -- | 0.91 (0.81-1.02) | 0.102 |

## Table Sx: Risk Factor Effects on CKD Within Each Cohort

|  |  | **Control Cohort** | | **Lupus Cohort** | |
| --- | --- | --- | --- | --- | --- |
| **Risk Factor** | **Comparison** | **HR (95% CI)** | **P-value** | **HR (95% CI)** | **P-value** |
| **Sex** | Female vs Male | 0.64 (0.58–0.71) | <0.001 | 1.04 (0.50–2.19) | 0.913 |
| **Age** | ≥60 years vs <60 years | 20.71 (18.49–23.19) | <0.001 | 13.42 (7.56–23.83) | <0.001 |
| **Socioeconomic Status** | Medium vs Low | 1.93 (1.72–2.17) | <0.001 | 1.72 (0.82–3.61) | 0.152 |
|  | High vs Low | 2.27 (1.99–2.59) | <0.001 | 4.16 (1.88–9.19) | <0.001 |
| **BMI** | BMI ≥30 vs BMI <30 | 1.86 (1.71–2.01) | <0.001 | 2.15 (1.26–3.67) | 0.005 |
| **Smoking** | Ever smoker vs Never smoker | 1.66 (1.52–1.82) | <0.001 | 1.33 (0.73–2.41) | 0.353 |
| **Diabetes** | Diabetes vs No diabetes | 4.44 (4.10–4.80) | <0.001 | 2.82 (1.65–4.82) | <0.001 |
| **Hypertension** | Hypertension vs No hypertension | 13.51 (12.10–15.08) | <0.001 | 8.48 (4.59–15.67) | <0.001 |

# Sensitivity analysis

## Competing Risks Analysis – Full Cohort

|  | **Cause-Specific Cox** | | | | **Fine-Gray** | | | |
| --- | --- | --- | --- | --- | --- | --- | --- | --- |
|  | **Unadjusted** | | **Adjusted** | | **Unadjusted** | | **Adjusted** | |
| **Outcome** | **CS-HR (95% CI)** | **P-value** | **CS-HR (95% CI)** | **P-value** | **FG-SHR (95% CI)** | **P-value** | **FG-SHR (95% CI)** | **P-value** |
| CKD | 2.05 (1.58-2.64) | <0.001 | 1.96 (1.50-2.54) | <0.001 | 1.98 (1.53-2.56) | <0.001 | 1.81 (1.23-2.64) | 0.002 |
| ESKD | 4.41 (2.06-9.42) | <0.001 | 3.13 (1.38-7.08) | 0.006 | 4.18 (1.96-8.94) | <0.001 | 2.89 (1.11-7.52) | 0.029 |
| MACE | 1.35 (1.08-1.67) | 0.007 | 1.63 (1.31-2.04) | <0.001 | 1.28 (1.03-1.60) | 0.024 | 1.40 (1.11-1.77) | 0.005 |
| All-Cause Mortality | 3.04 (2.51-3.70) | <0.001 | 4.52 (3.71-5.51) | <0.001 | N/A | N/A | N/A | N/A |
| CS-HR = Cause-Specific Hazard Ratio; FG-SHR = Fine-Gray Subdistribution Hazard Ratio; CI = Confidence Interval | | | | | | | | |
| Cause-specific models treat death as censoring. Fine-Gray models account for death as competing event. | | | | | | | | |
| MACE events include only those occurring ≥180 days (6 months) after index date. | | | | | | | | |
| Adjusted models control for: gender, age, baseline eGFR, BMI, smoking, diabetes, and hypertension. N = 92825; Adjusted Fine-Gray models N = 90766 (complete cases).  Comparison of cause-specific hazard ratios and Fine-Gray subdistribution hazard ratios. Cause-specific HRs treat death as censoring, while Fine-Gray SHRs account for death as competing event. MACE events include only those occurring ≥6 months after index date to exclude events potentially related to acute diagnosis period. N/A indicates mortality has no competing risk (reference outcome). | | | | | | | | |

## Table SX Baseline Characteristics analysis – Subpopulation with at least one protein tests after index

| **Characteristic** | **N** | **Control, N = 31,971^1^** | **Lupus, N = 801^1^** | **Difference^2^** | **p-value^3^** |
| --- | --- | --- | --- | --- | --- |
| Socioeconomic Status | 30,376 |  |  | 0.03 | 0.8 |
| Low |  | 7,848 (26%) | 207 (28%) |  |  |
| Medium |  | 17,146 (58%) | 425 (57%) |  |  |
| High |  | 4,635 (16%) | 115 (15%) |  |  |
| Age at index (years) | 32,772 | 55 (15) | 45 (16) | 0.67 | <0.001 |
| Female sex | 32,772 |  |  | 0.05 | 0.2 |
| Female |  | 26,905 (84%) | 687 (86%) |  |  |
| Male |  | 5,066 (16%) | 114 (14%) |  |  |
| Ethnicity | 19,325 |  |  | 0.12 | 0.007 |
| Arab |  | 6,328 (34%) | 195 (39%) |  |  |
| Jewish |  | 12,502 (66%) | 300 (61%) |  |  |
| Baseline Protein Level (mg/L) (within the first year from index date) | 1,456 | 235 (643) | 182 (362) | 0.10 | 0.2 |
| Ever smoker | 32,772 | 6,291 (20%) | 149 (19%) | 0.03 | 0.4 |
| Body mass index (kg/m²) | 32,319 | 29.1 (6.1) | 26.2 (5.9) | 0.48 | <0.001 |
| Baseline eGFR (mL/min/1.73m²) | 32,772 | 97 (17) | 104 (22) | -0.35 | <0.001 |
| Diabetes mellitus | 32,772 | 13,926 (44%) | 144 (18%) | 0.58 | <0.001 |
| Hypertension | 32,772 | 17,071 (53%) | 276 (34%) | 0.39 | <0.001 |
| Congestive Heart Failure (CHF) | 32,772 | 2,071 (6.5%) | 53 (6.6%) | -0.01 | 0.9 |
| Cerebrovascular accident (CVA) | 32,772 | 3,167 (9.9%) | 76 (9.5%) | 0.01 | 0.7 |
| Ischemic Heart Disease (IHD) | 32,772 | 6,757 (21%) | 165 (21%) | 0.01 | 0.7 |
| Hydroxychloroquine | 32,772 | 596 (1.9%) | 682 (85%) | -3.1 | <0.001 |
| Azathioprine | 32,772 | 329 (1.0%) | 206 (26%) | -0.78 | <0.001 |
| Mycophenolate mofetil | 32,772 | 110 (0.3%) | 50 (6.2%) | -0.34 | <0.001 |
| Methotrexate | 32,772 | 1,008 (3.2%) | 226 (28%) | -0.73 | <0.001 |
| Prednisone | 32,772 | 7,983 (25%) | 535 (67%) | -0.92 | <0.001 |
| Biologics (rituximab or belimumab) | 32,772 | 260 (0.8%) | 171 (21%) | -0.69 | <0.001 |
| Calcineurin inhibitors | 32,772 | 357 (1.1%) | 30 (3.7%) | -0.17 | <0.001 |
| RAAS inhibitors | 32,772 | 15,851 (50%) | 225 (28%) | 0.45 | <0.001 |
| Statins | 32,772 | 20,529 (64%) | 265 (33%) | 0.66 | <0.001 |
| Aspirin | 32,772 | 232 (0.7%) | 5 (0.6%) | 0.01 | 0.7 |
| Antihypertensives (BB/CCB/diuretics) | 32,772 | 18,420 (58%) | 386 (48%) | 0.19 | <0.001 |
| Diabetes medications | 32,772 | 14,808 (46%) | 170 (21%) | 0.55 | <0.001 |
| ^1^n (%); Mean (SD) | | | | | |
| ^2^Standardized Mean Difference | | | | | |
| ^3^Pearson's Chi-squared test; Welch Two Sample t-test; Fisher's exact test  Data are presented as mean (SD) for continuous variables and n (%) for categorical variables. Subpopulation includes patients with at least one proteinuria measurement (PCR or ACR) after index date. SMD = standardized mean difference; CHF = Congestive Heart Failure; CVA = Cerebrovascular accident; IHD = Ischemic Heart Disease; RAAS = Renin-Angiotensin-Aldosterone System; BB = Beta-blockers; CCB = Calcium Channel Blockers. Total subpopulation: 32772 (801 lupus, 31971 controls). | | | | | |

## Outcome analysis – Subpopulation with at least one protein tests after index

### Table SX: Cox Regression

|  | **Frequencies** | | | **Unadjusted** | | | **Adjusted** | | | |
| --- | --- | --- | --- | --- | --- | --- | --- | --- | --- | --- |
| **Outcome** | **Control, N = 91,681^1^** | **Lupus, N = 1,145^1^** | **p-value^1^** | **HR (95% CI)^2^** | **95% CI^2^** | **p-value** | **HR (95% CI)^2^** | **95% CI^2^** | **p-value** | |
| CKD | 346 (1.1%) | 14 (1.7%) | 0.074 | 1.80 | 1.06, 3.08 | 0.031 | 1.94 | 1.06, 3.53 | 0.031 | |
| ESKD | 104 (0.3%) | 7 (0.9%) | 0.019 | 3.05 | 1.42, 6.56 | 0.004 | 2.55 | 1.03, 6.34 | 0.044 | |
| MACE | 3,645 (11%) | 59 (7.4%) | <0.001 | 0.69 | 0.54, 0.90 | 0.005 | 1.24 | 0.95, 1.61 | 0.11 | |
| Mortality | 1,471 (4.6%) | 51 (6.4%) | 0.019 | 1.59 | 1.20, 2.10 | 0.001 | 3.74 | 2.81, 4.98 | <0.001 | |
| ^1^Pearson's Chi-squared test; Fisher's exact test, n (%)  ^2^HR = Hazard Ratio, CI = Confidence Interval  Subpopulation includes only patients with at least one protein measurement after index date, N = 32772. | | | | | | | | | |  |

## Multivariable models - Subpopulation

### *Table SX*A: Multivariable Cox Regression - CKD (per GFR<60 AND Protein>500) - Subpopulation

| **Variable** | **N** | **Event N** | **HR^1^** | **95% CI^1^** | **P-value** |
| --- | --- | --- | --- | --- | --- |
| **Lupus (vs Control)** | 32,319 | 357 | 1.94 | 1.06, 3.53 | **0.031** |
| **Female sex** |  |  |  |  |  |
| Female | 27,204 | 258 | — | — |  |
| Male | 5,115 | 99 | 1.33 | 1.05, 1.69 | **0.020** |
| **Age at index (years)** | 32,319 | 357 | 1.00 | 0.99, 1.01 | 0.6 |
| **Baseline eGFR (mL/min/1.73m²)** | 32,319 | 357 | 0.93 | 0.93, 0.94 | **<0.001** |
| **Body mass index (kg/m²)** | 32,319 | 357 | 1.00 | 0.98, 1.02 | >0.9 |
| **Ever smoker** | 32,319 | 357 | 1.32 | 1.05, 1.68 | **0.020** |
| **Diabetes mellitus** | 32,319 | 357 | 2.58 | 2.02, 3.29 | **<0.001** |
| **Hypertension** | 32,319 | 357 | 4.89 | 3.12, 7.64 | **<0.001** |
| ^1^HR = Hazard Ratio, CI = Confidence Interval | | | | | |

### Table S*X*B: Multivariable Cox Regression - ESKD - Subpopulation

| **Variable** | **N** | **Event N** | **HR^1^** | **95% CI^1^** | **P-value** |
| --- | --- | --- | --- | --- | --- |
| **Lupus (vs Control)** | 32,319 | 107 | 2.55 | 1.03, 6.34 | **0.044** |
| **Female sex** |  |  |  |  |  |
| Female | 27,204 | 76 | — | — |  |
| Male | 5,115 | 31 | 1.22 | 0.79, 1.89 | 0.4 |
| **Age at index (years)** | 32,319 | 107 | 0.96 | 0.94, 0.98 | **<0.001** |
| **Baseline eGFR (mL/min/1.73m²)** | 32,319 | 107 | 0.95 | 0.94, 0.96 | **<0.001** |
| **Body mass index (kg/m²)** | 32,319 | 107 | 0.97 | 0.94, 1.01 | 0.14 |
| **Ever smoker** | 32,319 | 107 | 1.59 | 1.04, 2.42 | **0.031** |
| **Diabetes mellitus** | 32,319 | 107 | 2.67 | 1.72, 4.15 | **<0.001** |
| **Hypertension** | 32,319 | 107 | 6.31 | 3.15, 12.6 | **<0.001** |
| ^1^HR = Hazard Ratio, CI = Confidence Interval | | | | | |

### Table S*X*C: Multivariable Cox Regression - MACE - Subpopulation

| **Variable** | **N** | **Event N** | **HR^1^** | **95% CI^1^** | **P-value** |
| --- | --- | --- | --- | --- | --- |
| **Lupus (vs Control)** | 32,319 | 3,670 | 1.24 | 0.95, 1.61 | 0.11 |
| **Female sex** |  |  |  |  |  |
| Female | 27,204 | 2,542 | — | — |  |
| Male | 5,115 | 1,128 | 2.01 | 1.87, 2.17 | **<0.001** |
| **Age at index (years)** | 32,319 | 3,670 | 1.04 | 1.03, 1.04 | **<0.001** |
| **Baseline eGFR (mL/min/1.73m²)** | 32,319 | 3,670 | 1.00 | 0.99, 1.00 | **0.032** |
| **Body mass index (kg/m²)** | 32,319 | 3,670 | 1.00 | 1.00, 1.01 | 0.2 |
| **Ever smoker** | 32,319 | 3,670 | 1.40 | 1.30, 1.50 | **<0.001** |
| **Diabetes mellitus** | 32,319 | 3,670 | 1.57 | 1.46, 1.68 | **<0.001** |
| **Hypertension** | 32,319 | 3,670 | 2.35 | 2.15, 2.58 | **<0.001** |
| ^1^HR = Hazard Ratio, CI = Confidence Interval | | | | | |

### Table S*X*D: Multivariable Cox Regression - All-Cause Mortality - Subpopulation

| **Variable** | **N** | **Event N** | **HR^1^** | **95% CI^1^** | **P-value** |
| --- | --- | --- | --- | --- | --- |
| **Lupus (vs Control)** | 32,319 | 1,499 | 3.74 | 2.81, 4.98 | **<0.001** |
| **Female sex** |  |  |  |  |  |
| Female | 27,204 | 1,103 | — | — |  |
| Male | 5,115 | 396 | 1.44 | 1.28, 1.62 | **<0.001** |
| **Age at index (years)** | 32,319 | 1,499 | 1.10 | 1.09, 1.11 | **<0.001** |
| **Baseline eGFR (mL/min/1.73m²)** | 32,319 | 1,499 | 1.00 | 0.99, 1.00 | 0.4 |
| **Body mass index (kg/m²)** | 32,319 | 1,499 | 1.01 | 1.00, 1.02 | **0.016** |
| **Ever smoker** | 32,319 | 1,499 | 1.38 | 1.23, 1.55 | **<0.001** |
| **Diabetes mellitus** | 32,319 | 1,499 | 1.62 | 1.45, 1.81 | **<0.001** |
| **Hypertension** | 32,319 | 1,499 | 1.33 | 1.15, 1.54 | **<0.001** |
| ^1^HR = Hazard Ratio, CI = Confidence Interval | | | | | |

Supplementary Figure S1


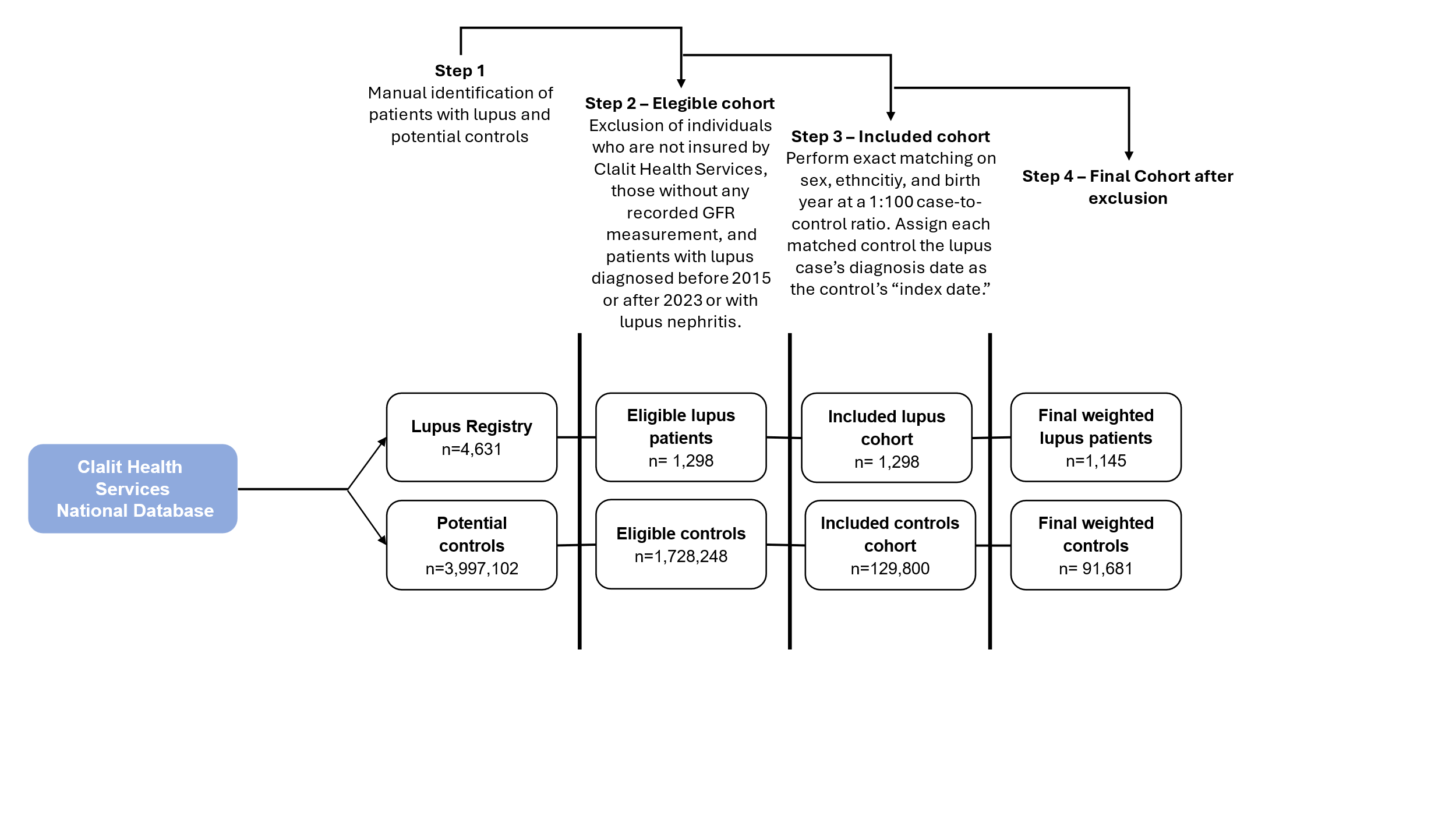


Supplementary Figure S2


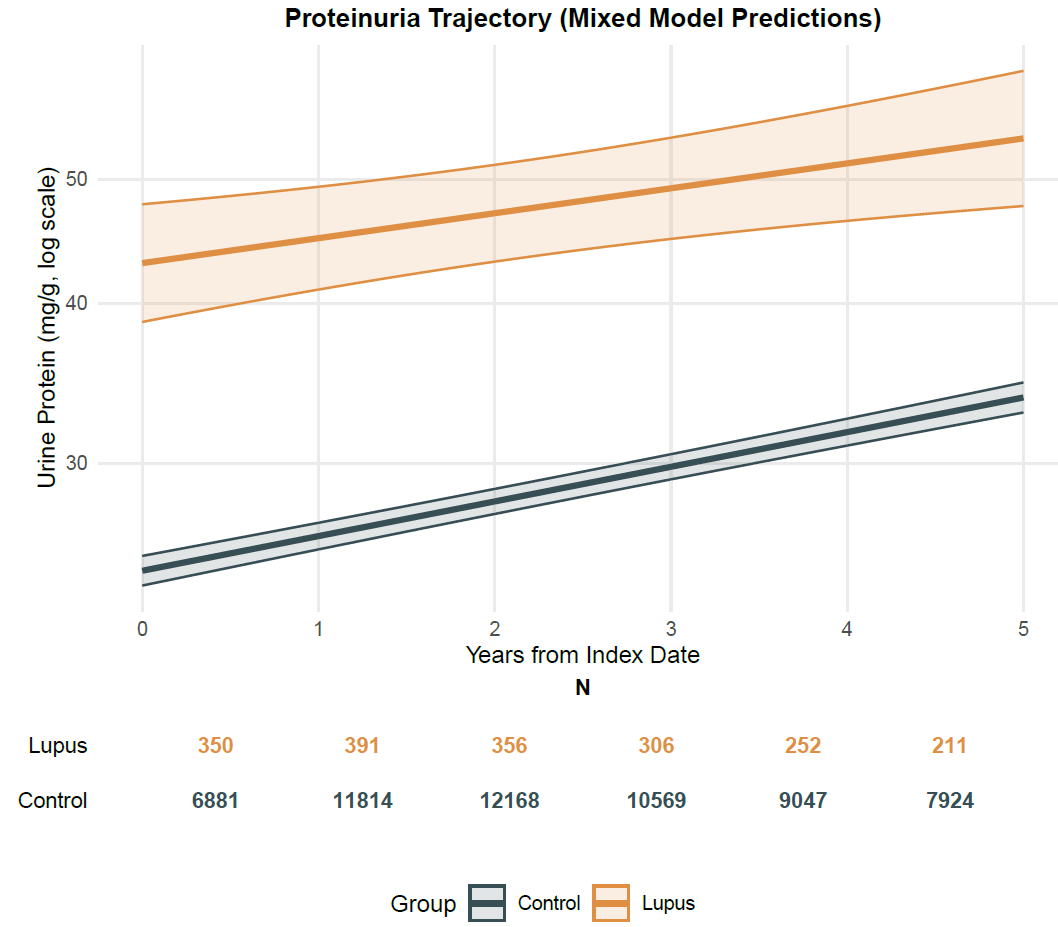


Interaction between SLE and traditional risk factors for CKD risk (Supplementary Figure S3)


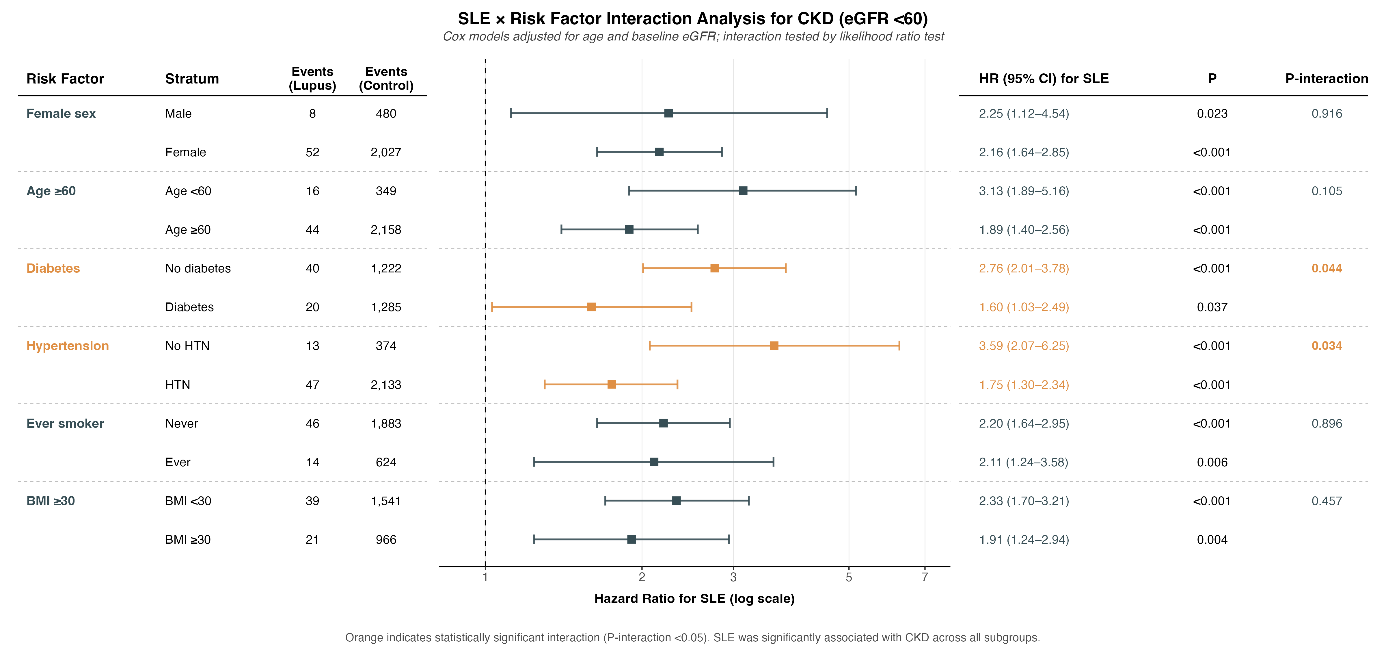

Supplement: keag307_Supplementary_Data [file keag307_supplementary_data.docx]
